# Nasal Swell Body Characteristics in Patients With Septal Perforation

**DOI:** 10.1002/oto2.43

**Published:** 2023-02-23

**Authors:** Daniela A. Brake, Sam Snider, Amar Miglani, Grant S. Hamilton, Stephen F. Bansberg

**Affiliations:** ^1^ Department of Otolaryngology–Head and Neck Surgery Mayo Clinic Phoenix Arizona USA; ^2^ Department of Otolaryngology–Head and Neck Surgery Mayo Clinic Rochester Minnesota USA

**Keywords:** nasal obstruction, nasal swell body, septal perforation, septal perforation repair, septal swell body

## Abstract

**Objective:**

To determine whether septal perforations have an effect on nasal swell body (NSB) size.

**Study Design:**

Retrospective cohort study.

**Setting:**

Two tertiary academic medical centers.

**Methods:**

Computed tomography maxillofacial scans of 126 patients with septal perforation and 140 control patients from November 2010 to December 2020 were evaluated. Perforation etiology was determined. Measurements included perforation length and height and swell body width, height, and length. Swell body volume was calculated.

**Results:**

The width and volume of the NSB are significantly smaller in perforation patients when compared to controls. The swell body is significantly smaller and thinner in perforations exceeding 14 mm in height compared to small perforations. Perforation etiology groupings into prior septal surgery, septal trauma, septal inflammatory, and mucosal vasoconstriction categories all demonstrated decreased swell body volume and width compared to controls. Inflammatory etiology had the greatest decrease in swell body size. The hemi‐swell body on the contralateral side of a septal deviation is significantly thicker than the ipsilateral side.

**Conclusion:**

The NSB is smaller in patients with septal perforation regardless of perforation size or etiology.

The nasal swell body (NSB), also known as Kiesselbach's body, septal turbinate, septal swell body, or intumescentia septi nasi anterior, is an area of the nasal septum anterior to the middle turbinate and superior to the inferior turbinate.^1^, ^2^, ^3^, ^4^ The mucosa of the NSB is thickened compared to the remainder of the septal mucosa. Structurally, the underlying quadrangular cartilage is often thicker than the surrounding cartilage. The NSB has an ellipsoid shape approximately 3 cm in length by 2 cm in height as determined by imaging and cadaveric studies. The width of the septum through the NSB is approximately 1 cm. Clinically, the NSB can be mistaken for a high septal deviation.

The NSB is believed to be a dynamic structure with the ability to modulate nasal airflow.^1^, ^4^ Histological studies have compared the NSB to the inferior turbinate. Wexler et al analyzed 2 to 4 mm biopsies of the NSB and inferior turbinate in 14 adults undergoing septoplasty.^5^ The NSB contained 49.9% seromucinous glands compared to 19.9% for the inferior turbinate, with only a 10% versus 28% composition of venous sinusoids. The study concluded the NSB was comprised of highly glandular tissue with a primary secretory mucosal moisturizing versus nasal airflow regulation function. More recently, Costa et al studied the NSB using MRI on 54 patients and histological examination on 10 full‐thickness cadaveric specimen biopsies.^2^ They noted a significantly higher proportion of venous sinusoid composition and a smaller proportion of glandular composition compared to the Wexler study and suggested the sinusoids found in deeper areas sampled in their study accounted for the difference. The high proportion of venous sinusoids implied a capacity for dynamic nasal airflow alteration. The anterior extent of the NSB can reach the nasal valve angle.^6^ The NSB is anatomically positioned and functionally designed to regulate nasal airway resistance and humidify the nasal cavity.

The growing interest in the NSB and its impact on nasal obstruction and patient quality of life has produced studies on swell body size in the presence of chronic rhinosinusitis, allergic rhinitis, and septal deviation. Setlur and Goyal demonstrated that NSB hypertrophy occurs contralateral to a septal deformity.^7^ Hypertrophy thickness correlated with the degree of deformity. The NSB has been found to be thicker in patients with allergic rhinitis and chronic rhinosinusitis.^3^, ^8^ Swell body surgical reduction has emerged as a promising treatment for patients with nasal obstruction and corroborative findings on physical examination.^9^, ^10^, ^11^, ^12^

A perforation of the nasal septum may border the swell body and, with larger defects, erode into it (Figure 1). Septal mucosal inflammation or an alteration in nasal airflow physiology related to the perforation or its etiology could effect NSB size and symptom presentation. Characterization of the NSB in perforation patients may have clinical implications considering closure utilizing nasal mucosal flaps.^13^ The primary goal of this study was to determine whether there are changes in NSB size in the presence of a septal perforation.

**Figure 1:**
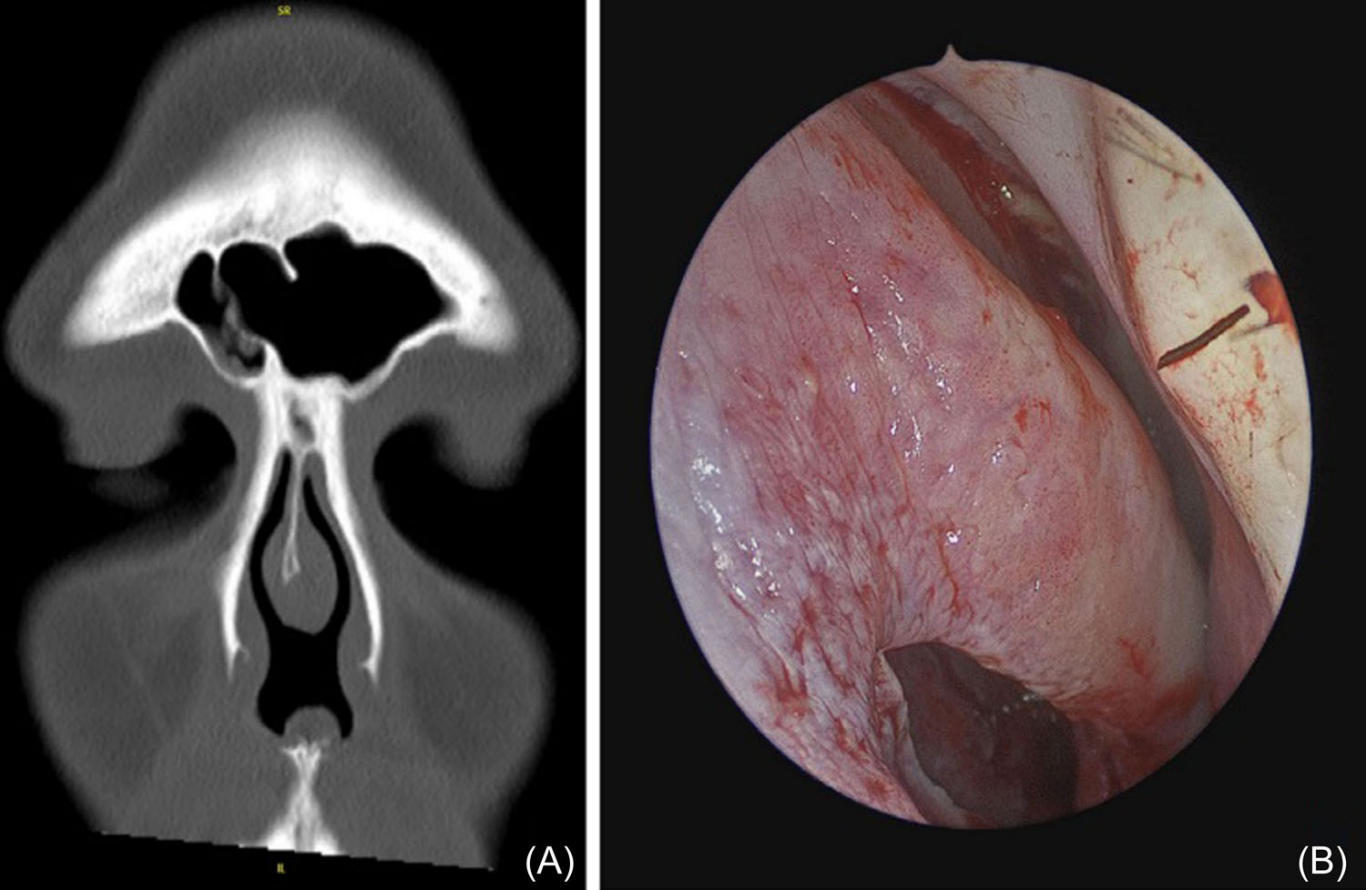
(A) Sinus computed tomography scan demonstrating a thick NSB opposite a high right‐sided deviation superior to a perforation. (B) Intraoperative endoscopic photo of the same patient demonstrating the superior mucosal flap containing the NSB. NSB, nasal swell body.

## Materials and Methods

This study was approved by the Mayo Clinic Institutional Review Board (IRB #19‐010429). A retrospective review of computed tomography (CT) scans was performed of patients with septal perforation and compared to a control (no perforation present) patient population. The randomized selection of controls was performed by selecting every 10th scan from a comprehensive, chronological list of patients who underwent thin‐cut CT sinus or maxillofacial scans at Mayo Clinic Arizona or Minnesota sites. The control cohort was obtained from CT scans performed from January 2012 to December 2020. Scans were excluded from the control cohort if there was evidence of facial trauma, sinonasal tumor, sinusitis, or another sinonasal condition obscuring demarcation and accurate measurement of the NSB. Patients with perforation were selected from pretreatment scans performed for nonconsecutive patients who underwent attempted perforation repair in Arizona or for fabrication of a customized silastic septal (button) prosthesis in Minnesota from November 2010 to February 2020.

Patient demographic information was collected from the medical record. Perforation etiology, history of septal surgery, and concurrent inflammatory conditions were noted. Etiologies were grouped into 5 categories to facilitate statistical analysis. Perforation length and height were measured in millimeters off the CT scan. Coronal, sagittal, and transverse scan views were utilized as indicated for measuring. For this study, perforations were classified by vertical height as small (<5 mm), medium (5‐14 mm), and large (>14 mm). Measurements were made to determine the septal positioning of the perforation related to the NSB. Lund‐Mackey scores were recorded for both control and perforation scans. The presence of a superior septal deformity was determined in control patients. The septal deviation was defined as being greater than 5 degrees from a line between the maxillary crest and the superior septal/upper lateral cartilage junction. Swell body width and length for the total, right, and left hemi‐NSBs were measured in all scans. Total swell body volume was calculated using ellipsoid volume as a proxy.

Statistical analysis was conducted using Student's *t* test with a 1‐tailed distribution and 2‐sample unequal variance for comparison between groups, and 1‐way analysis of variance (ANOVA) for comparing multiple groups with significance noted if *p* < .05. Pearson's correlation coefficient was calculated to determine the relationship of NSB size with age, perforation characteristics with NSB, vertical perforation height and NSB volume, and for the relationship of hemi‐NSB volume and degree of septal deviation. The software used for statistical analysis was Microsoft® Excel® Data Analysis Toolpak.

## Results

The CT scans of 126 perforation patients and 140 controls were evaluated for this study. Seventy‐five patients with a perforation obtained a scan for the fabrication of a customized septal button prosthesis and 51 were scanned prior to attempted surgical closure. The control patient average age was 57.4 (range 20‐95) years and 57.1% were female. Eleven (7.9%) of control patients underwent prior septal surgery. Perforation patient average age was 53.4 (range 13‐84) years and 58.7% were female. The most common perforation etiology was septal surgery (38.1%). Etiology could not be determined in 33 (26.2%) patients. Perforation etiology grouped into 5 categories is noted in Table 1. The mean perforation length was 14.8 (range 1.8‐41.8) mm and height was 10.6 (range 1.8‐27.8) mm. Perforation size groupings based on vertical height into small (<5 mm), medium (5‐14 mm), and large (>14 mm) are also noted in Table 1. The mean distance from the NSB epicenter to the closest perforation margin for small, medium, and large perforations was 11.6, 8.9, and 6.1 mm, respectively. The mean Lund‐Mackey score in controls was 2.7 (range 0‐14) and 4.3 (range 0‐17) in perforation patients (*p* = .002). Allergic rhinitis prevalence was 37.8% in controls and 40.5% in perforation patients (*p* < .001).

**Table 1:** Perforation Etiology and Size Category

	Perforation patients (**%**)	Controls (**%**)
N (patients)	**126 (47.4)**	**140 (52.6)**
Perforation etiology		
Septal surgery	48 (38)	
Indeterminate	29 (23.0)	
Trauma	22 (17.5)	
Digital/behavioral		
Cauterization		
Steroid spray		
Foreign body		
Systemic Inflammatory	13 (10.3)	
Autoimmune		
Vasculitic		
Mucosal vasoconstrictive	14 (11.1)	
Cocaine		
Decongestant spray		
Size category (mm)		
Small (<5)	19 (15.1)	
Medium (5‐14)	86 (68.2)	
Large (>14)	21 (16.7)	

NSB width and volume measurements in control and perforation patients are noted in Table 2. Septal perforation patients overall demonstrated significantly thinner (*p* < .001) and smaller volume (*p* < .001) NSBs than controls. In control patients, male patients had a significantly wider NSB (*p* < .006) and volume (*p* < .001) compared to females. Likewise, in perforation patients, males demonstrated significantly wider (*p* < .002) and increased volume (*p* < .001) NSBs compared to females. There was no difference in gender predominance between groups (*p* = .06). Patient age did not significantly effect NSB width (*p* = .22) or volume (*p* = .18) as measured in the control group. There was no significant difference in NSB width (*p* = .76) or volume (*p* = .79) in the perforation cohort correlated to age. Swell body width and volume were correlated to perforation etiology. There was no significant difference in NSB volume among the different etiology groupings (Table 2). Likewise, there was no significant difference in NSB width by etiology grouping by ANOVA analysis. Patients with a perforation following prior septal surgery demonstrated the largest mean NSB volume while there was a trend toward decreased NSB volume with perforations due to an inflammatory process.

**Table 2:** Nasal Swell Body Volume and Width in Perforation Patients Versus Controls

	Perforation patients	Controls	*p*
NSB total mean volume (mm^3^)	1957	2617	<.001
Septal surgery	2193		
Indeterminate	1851		
Trauma	1911		
Systemic inflammatory	1390		
Mucosal vasoconstrictive	1945		
Females	1675	2363	
Males	2367	2947	
	*p* < .001	*p* < .001	
Average total NSB width (mm)	9.97	11.2	<.001
Septal surgery	10.0		
Indeterminate	10.3		
Trauma	10.1		
Systemic inflammatory	9.3		
Mucosal vasoconstrictive	10.2		
Females	9.5	10.9	
Males	10.7	11.7	
	*p* < .002	*p* < .006	

Abbreviation: NSB, nasal swell body.

**Figure 2:**
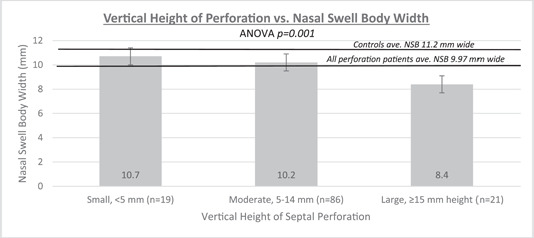
Vertical height of perforation versus nasal swell body width. Perforation measurements are classified into 3 groups based on the height of the perforation and compared based on average nasal swell body width. ANOVA, analysis of variance; NSB, nasal swell body.

**Figure 3:**
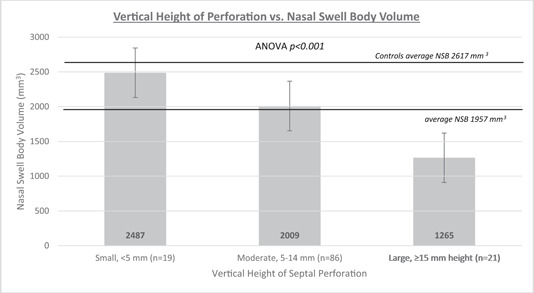
Vertical height of perforation versus nasal swell body volume. Perforation measurements are classified into 3 groups based on the height of the perforation and compared based on average nasal swell body volume. ANOVA, analysis of variance; NSB, nasal swell body.

Swell body width and volume compared to perforation size are noted in Figures 2 and 3. The NSB volume in patients with large perforations is significantly smaller compared to patients with medium size (*p* < .001) and small (*p* < .001) perforations. Medium‐size perforations are also associated with a significantly smaller NSB volume than small perforations (*p* = .046). Swell body volume demonstrated a negative, weak correlation as perforation vertical height increased (Pearson's correlation coefficient *r* = −.15), meaning as the vertical height of the perforation increases, the NSB volume decreases. Swell body volume had a negative, moderately strong correlation as the perforation area increased and the perforation margin advanced superiorly (Pearson's correlation coefficient *r* = −.35). As the perforation area enlarges, the swell body volume decreases.

A superior septal deviation was present in 52.0% of the CT scans of control patients. The average angle of the deviation was 8.4°. The hemi‐NSB on the contralateral side of a superior septal deviation was significantly thicker than the hemi‐NSB on the side of the deviation (6.2 vs 5.0 mm, *p* < .001).

## Discussion

Nasal septal perforations represent a heterogenous group of disorders as determined by symptoms, etiology, size, mucosal condition, concurrent rhinologic conditions, and the presence of aesthetic deformity. Most symptomatic perforations involve the anterior cartilaginous septum and are likely to enlarge over time. Erosion of the perforation into the NSB is another variable that could affect perforation symptomatology or treatment outcomes. We hypothesized various factors could increase the size of the NSB when a perforation is present. A perforation due to an inflammatory condition or topical exposure, or an inflamed and crusted perforation margin due to chondritis associated with exposed cartilage, could thicken the NSB. The swell body septal turbinate could hypertrophy consequent to altered nasal airflow physiology in the presence of a perforation.

**Figure 4:**
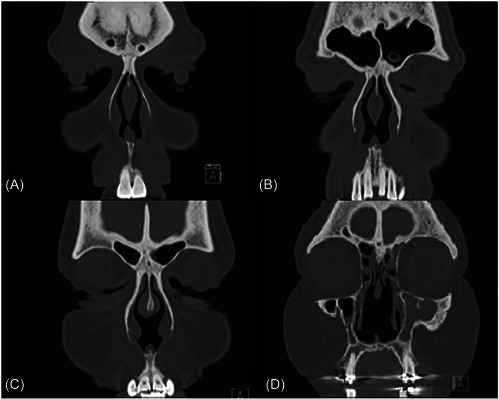
Coronal CT images depicting representative nasal septal perforations from each of 3 size groups (A, small; B, moderate; and C, D, large). Swell body visible immediately superior to perforation. CT, computed tomography.

A prior radiographic study determined the epicenter of the fusiform‐shaped NSB to be 2.5 cm from the nasal floor.^2^ Septal mucosa specimen mapping found the distance from the nasal floor to the NSB inferior boundary ranged between 1.8 and 2.6 cm.^1^ For this study perforations were classified into small, medium, and large based on vertical height and distance to the NSB epicenter measurements. Size classification for this study was chosen such that small perforations would have minimal if any, contact with the NSB inferior border while large perforations would likely erode into or through the NSB (Figure 4). Sixty‐eight percent of the perforations were classified as medium‐sized and brought focus to the perforation‐swell body interface. This study found that, overall, NSB volume and thickness decreased in perforated septums compared to controls. Medium‐sized perforations were not associated with a larger NSB. Not unexpectedly, erosion into the NSB effecting both volume and thickness was more pronounced as perforations enlarge superiorly.

Rather than enlarging to compensate for the loss of septal tissue, the results of this study suggest the NSB may be subject to the same destructive processes that cause the perforation. Perforation etiology classification to facilitate data analysis was guided by the mechanism of septal injury. Patients with perforations related to an inflammatory process demonstrated the greatest reduction in NSB volume, possibly due to widespread submucosal injury, scaring, and atrophy occurring over time following acute inflammation. Statistical analysis comparing NSB volume by perforation etiology demonstrated NSBs with a perforation due to an inflammatory process trended toward a much smaller volume compared to other etiologies. The inability to detect statistical significance between inflammatory and other etiologies was likely due to the small sample size of the inflammatory group. Swell bodies in patients with perforations due to septal surgery were larger, suggesting mucoperichondrial elevation has less effect on this structure than other perforation etiologies. Changes in NSB size correlated to extent of mucoperichondrial or periosteal elevation and cartilage/bone removal is a subject for further study. Perforations attributed to septal trauma and those of indeterminant etiology demonstrated an intermediate decrease in NSB size.

Increased NSB size in the presence of chronic rhinosinusitis and allergic rhinitis has been reported.^3^, ^8^, ^14^ Lund‐Mackey scores and incidence of allergic rhinitis were higher in our perforation cohort and may have resulted in an NSB size difference that was less than if the 2 cohorts were matched by the degree of rhinitis and sinusitis.

The NSB appears to play a role in the regulation of nasal airflow. Septal perforations disturb nasal airflow patterns.^15^, ^16^ Computational fluid dynamics (CFD) technology has emerged as an important tool for studying nasal physiology. CFD has demonstrated impairment of the nasal warming function in perforated noses, and this is most notable for larger, anterior perforations.^17^, ^18^, ^19^ Diminished mucosal function associated with a smaller NSB in the perforated nose may further impact nasal physiology and contribute to the dryness, crusting, bleeding, and congestion prevalent in these patients. A possible decrease in glandular elements could further impact mucosal moisturizing. Histologic study of the NSB of perforated septums which quantifies glandular elements and vasoactive sinusoids may provide further insight into the pathophysiology associated with septal perforations.

An often‐stated advantage of perforation repairs utilizing nasal mucosa is the physiologic nature of the repair. Longitudinally‐oriented flaps developed from the superior septum and nasal floor are frequently utilized to achieve tension‐free perforation closure. The senior author has attempted over 400 endonasal perforation repairs utilizing bilateral, longitudinally‐oriented nasal mucosal flaps supported with an autogenous tissue interposition graft.^20^ Though a thick swell body may improve repair durability, varied degrees of persistent nasal obstruction have been noted following many successful closures. An inferiorly advanced flap from the superior septum containing the NSB can be the cause of persistent postoperative obstruction.^13^ Secondary surgical thinning of the repair improves symptom outcomes. Attempting to improve our understanding of this clinical experience provided the impetus for this study. The possibility of persistent postoperative obstruction is an inherent disadvantage of perforation repairs using flaps that incorporate NSB tissue into them. This study found the NSB in the perforated septum when compared to a control cohort is not pathologically enlarged to increase the probability of obstruction.

Many of the nasal flap perforation repair techniques described utilize mucosa superior to defect through the development of a longitudinally‐oriented bipedicled advancement flap. Unilateral superior mucosal incision and inferior flap advancement mitigate against the possibility of bilateral superior cartilage exposure and devascularization with subsequent necrosis and reperforation. The superior flap, which can be extended to incorporate mucosa from the undersurface of the upper lateral cartilage, is the most consequential flap in our larger perforation repairs.^21^ The nasal obstruction noted postoperatively in a prior study using a unilateral, superior longitudinally‐oriented superior flap was noted to occur on the side of the superior flap.^13^ A secondary study interest analyzed the relationship between superior septal deviations and NSB thickness in the control cohort. Our findings corroborate those from a prior study that documented hemi‐NSB hypertrophy on the side contralateral to the direction of the septal deviation.^7^ We have not routinely obtained a CT scan prior to attempting perforation repair. Scan or physical examination findings of a high septal deviation with associated smaller NSB could be useful for surgical planning. A unilateral superior flap developed on the side of a smaller NSB would decrease the risk of persistent postoperative obstruction related to this technique‐driven repair asymmetry.

A gender difference in NSB size has not been previously reported, though an increased incidence of NSB in males was noted in a prior radiographic study.^14^ This study found a significant increase in NSB width and volume for males in both control and perforation patients. Significant differences detected in this study following data analysis are unlikely due to gender as there was no male predominance among the cohorts subjected to comparative statistical analyses. Swell body size was not correlated to age in either the control or perforation group.

A retrospective review using CT scans creates multiple study limitations. Information on clinical factors that could affect the swell body and findings of this study were not consistently collected in the perforation group to allow for study inclusion. The health of the periperforation mucosa, the extent of mucosal elevation and septal cartilage/bone removal in patients with prior septal surgery, the time period and intensity of topical exposures causing the perforation, and how long the perforation has been present are factors that could impact NSB size. Some patients have multiple potential etiologies. As noted previously, septal perforation represents a heterogenous condition with multiple variables confounding their assessment and treatment. Given the absence of preperforation CT scans and a detailed and standardized method of nasal evaluation for the patient with a perforated septum, a control group can provide relevant data for comparison study attempting to understand a postoperative obstruction condition we have noted in some patients with a successful repair.

This study was performed using nonconsecutive perforation patients presenting to 2 institutions with differences in their approach to perforation management. Selection for study inclusion was biased toward those patients who were symptomatic from their perforation. As this study included the spectrum of perforation size and etiologies, we believe selection bias has been minimized. We subjected the data from other size and etiology groupings to statistical analysis and found no significant difference in the study findings. Some control subjects presented with sinonasal symptoms with or without known pre‐existing sinus disease and, therefore, may not fully represent the general population. The differences in age and allergic rhinitis predominance between groups, although reaching statistical significance, were likely of little clinical importance, with a distinction of only about 4 years in age and a 3% difference in the rate of allergic rhinitis in light of a wide range of prevalence reported in the literature. They were included for NSB measurement as sinusitis is a frequent condition concurrent with a perforation.^22^

## Conclusion

This study found a decrease in NSB width and volume when a septal perforation is present. These findings were noted in perforations bordering the swell body and were greater for vertically larger perforations extending into the swell body. We also found the mucosa on the ipsilateral side of a high septal deformity, present in half of our control patients, is thinner relative to the contralateral side. The rhinologic surgeon interested in mucosal flap repair of the perforated septum should understand the possible effect of the swell body on surgical technique and outcomes.

## Author Contributions

**Daniela A. Brake**, design, conduct, acquisition and interpretation of data, analysis, drafting and critical revision, final approval of publication; **Sam Snider**, conduct, acquisition and interpretation of data, analysis, drafting and critical revision, final approval of publication; **Amar Miglani**, design, critical revision, final approval of publication; **Grant S. Hamilton III**, acquisition of data, critical revision, final approval of publication; **Stephen F. Bansberg**, conception, design, acquisition and interpretation of data, writing, critical revision, final approval of publication.

## Disclosures

**Competing interests:** None.

**Funding source:** None.
